# Systematic analysis of the polyphenol metabolome using the Phenol‐Explorer database

**DOI:** 10.1002/mnfr.201500435

**Published:** 2015-10-15

**Authors:** Joseph A. Rothwell, Mireia Urpi‐Sarda, Maria Boto‐Ordoñez, Rafael Llorach, Andreu Farran‐Codina, Dinesh Kumar Barupal, Vanessa Neveu, Claudine Manach, Cristina Andres‐Lacueva, Augustin Scalbert

**Affiliations:** ^1^ International Agency for Research on Cancer (IARC)Section of Nutrition and MetabolismBiomarkers GroupLyon CEDEXFrance; ^2^ Department of Nutrition and Food ScienceCampus TorriberaFaculty of PharmacyUniversity of BarcelonaBarcelonaSpain; ^3^ INRAUMR1019Human Nutrition UnitCRNH AuvergneClermont‐FerrandFrance

**Keywords:** Bioavailability, Intervention studies, Metabolism, Pharmacokinetics, Polyphenol metabolome, Pure polyphenol doses

## Abstract

**Scope:**

The Phenol‐Explorer web database details 383 polyphenol metabolites identified in human and animal biofluids from 221 publications. Here, we exploit these data to characterize and visualize the polyphenol metabolome, the set of all metabolites derived from phenolic food components.

**Methods and results:**

Qualitative and quantitative data on 383 polyphenol metabolites as described in 424 human and animal intervention studies were systematically analyzed. Of these metabolites, 301 were identified without prior enzymatic hydrolysis of biofluids, and included glucuronide and sulfate esters, glycosides, aglycones, and *O*‐methyl ethers. Around one‐third of these compounds are also known as food constituents and corresponded to polyphenols absorbed without further metabolism. Many ring‐cleavage metabolites formed by gut microbiota were noted, mostly derived from hydroxycinnamates, flavanols, and flavonols. Median maximum plasma concentrations (*C*_max_) of all human metabolites were 0.09 and 0.32 μM when consumed from foods or dietary supplements, respectively. Median time to reach maximum plasma concentration in humans (*T*_max_) was 2.18 h.

**Conclusion:**

These data show the complexity of the polyphenol metabolome and the need to take into account biotransformations to understand in vivo bioactivities and the role of dietary polyphenols in health and disease.

**Abbreviations**
*C*_max_: maximum plasma concentration
*T*_max_: time to reach maximum plasma concentration

## 1 Introduction

Polyphenols are a large and complex class of bioactive plant compounds. Around 500 are known to be consumed from human diets, and the compounds are further divided into numerous classes and subclasses according to their carbon skeleton 1 (Supporting Information 1). The average total intake of polyphenols per person in Western populations could be as high as 2 g/day 2. Polyphenol consumption may protect against cardiovascular diseases, cancers, and a range of other diseases 3, 4. As a result, the last 15 years has seen the accumulation of an expansive body of literature on their occurrence, metabolism, bioactivities, and bioavailability.

The total set of polyphenols or polyphenol metabolites present in foods or in human biospecimens is termed the “polyphenol metabolome” 5. Circulating metabolites are unlikely to be the same compounds as ingested polyphenols, but derivatives formed either by endogenous metabolism or by the microbiota, which allow effective transport and elimination from the body. Many of these metabolites have been identified in human and animal biofluids from controlled intervention studies with polyphenol‐rich foods or pure polyphenols. All metabolites described in the literature have been extracted and stored in Phenol‐Explorer, a web database originally conceived as a repository of polyphenol food composition data 6, 7. The database enables the retrieval of all known polyphenol metabolites formed upon uptake of a particular food or polyphenol with accompanying pharmacokinetic data, if available. Until the release of this database, the identities of polyphenol metabolites were scattered throughout a large volume of literature and frequently difficult to obtain.

Previous studies have reviewed polyphenol absorption and metabolism from controlled intervention studies, taking into account the dose size and mode of administration 8, 9. Instead, the aim of this study is to exploit all Phenol‐Explorer data to systematically analyze the polyphenol metabolome and, more specifically, the nature of the metabolites reported in intervention studies and their pharmacokinetic properties. Polyphenol metabolites are distributed to tissues and may act, e.g., by modulating signaling cascades, which govern biological processes, such as endothelial function 10 and cell‐cycle control 11. Thus a notable use of the database might be to find out which metabolites circulate after administration of a given polyphenol‐rich source that is suspected of imparting beneficial effects upon health 12. Qualitative and pharmacokinetic knowledge of the polyphenol metabolome has also been essential for the identification of biomarkers of polyphenol intake in metabolomic studies 13.

## 2 Materials and methods

### 2.1 Sources of data on polyphenol metabolism and pharmacokinetics

All polyphenol intervention studies in the Phenol‐Explorer database were included in the analysis 7. Publications were required to meet the following base criteria: (i) be intervention studies using a single or repeated orally administered dose of a normal food source, an experimental food (e.g., food extract, dried, and powdered foods) or an oral supplement; (ii) be conducted in vivo on disease‐free humans or animals; (iii) use an appropriate analytical technique capable of reliably identifying metabolites; (iv) detect or quantify at least one polyphenol metabolite in urine or plasma. In brief, separate “interventions” were identified from each publication, where one intervention was defined as the administration of a given dose of polyphenol source (or control) to a single species, with subsequent collection of biofluids. Study design details for each intervention were entered into the database followed by the identities of metabolites corresponding to each intervention, along with any pharmacokinetic details.

### 2.2 Data analysis

Phenol‐Explorer tables on metabolites detected, metabolite concentrations, plasma pharmacokinetics, and urine pharmacokinetics were exported and data analyzed using Microsoft Excel and R statistical software (http://www.R‐project.org/). Chemical similarities, based on Tanimoto scores, were calculated for each pair of Phenol‐Explorer metabolites using the PubChem structure clustering tool. The MetaMapp tool (http://metamapp.fiehnlab.ucdavis.edu) was used to format and filter the PubChem similarity output matrix, associating only pairs of metabolites with similarity scores >0.7 14. The matrix was mapped to a network graph using Cytoscape open‐source software 15. The metabolic transformations of pure compounds administered to humans and animals were visualized using Circos open‐source software 16.

## 3 Results

### 3.1 Polyphenol intervention studies

Data originate from 221 publications describing intervention studies with polyphenol‐rich sources. These publications produced 424 separate interventions, of which 398 were on humans or rats. Polyphenol sources were regular and “experimental” foods and included raw foods, processed foods, and food extracts. In addition, pure polyphenol doses were also administered in solution or in powder, tablet, or capsule form. Flavonoids were most studied overall, particularly the flavanones and anthocyanins. Foods and experimental foods were most commonly administered to humans, whereas pure compounds most commonly administered to rats. Doses were either given once or at time intervals, where intervention duration varied from a few hours to several months. The range of study designs is summarized in Table 1.

**Table 1:** Polyphenol intervention study design

	Number of intervention studies			
	Human	Rat	Other^a^	Total
Polyphenol subclass studied				
Anthocyanins	35	22	5	62
Flavanols	40	32	6	78
Flavanones	31	12	2	45
Flavonols	20	9	0	29
Hydroxybenzoic acids	24	13	4	41
Hydroxycinnamic acids	4	19	0	23
Isoflavones	38	13	2	53
Lignans	8	22	2	32
Stilbenes	25	3	5	33
Tyrosols	18	10	0	28
Duration				
Less than 12 h	42	22	5	69
12–24 h inclusive	115	58	7	180
2–7 days inclusive	41	21	1	63
1 month or more	45	54	13	112
Dose type				
Experimental food	101	49	12	162
Food	108	19	3	130
Pure compound	34	87	11	132
Repeated dose				
No	191	112	17	320
Yes	52	43	9	104

Four hundred twenty‐four separate interventions were included, originating from 221 publications.

a Other animal models used were mouse, pig, dog, and sheep. All references used can be obtained from http://www.phenol‐explorer.eu.

### 3.2 Diversity of polyphenol metabolites

**Figure 1:**
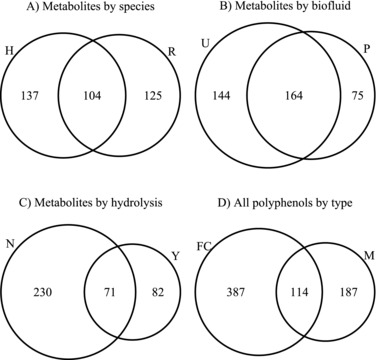
Venn representation of the polyphenol metabolome as described by Phenol‐Explorer (*n* = 383). (A) Metabolites identified in human (H) or rat (R) biofluids (other animal models omitted). (B) Metabolites from all species identified in urine (U) or plasma (P). (C) Metabolites identified without (N) or after (Y) hydrolysis of biospecimens. (D) Phenolic food components (FC) or metabolites (M) formed in humans or experimental animals.

A total of 383 polyphenol metabolites identified in blood or urine were compiled from the literature as part of the polyphenol metabolome. Similar numbers of metabolites were reported in human and rat studies, with 104 metabolites common to both species (Fig. 1A). Fewer metabolites were reported only in plasma than only in urine, and around half were identified in both biofluids (Fig. 1B). Most metabolites (*n* = 301) were identified without prior hydrolysis of the glucuronides and sulfate esters with enzymes (Fig. 1C). These included 53 glucuronides and 23 sulfate esters as well as 67 glycosides (Supporting Information 2). Anthocyanin glycosides accounted for most of glycoside conjugates. The remaining metabolites were aglycones or esters. Of the 301 metabolites identified in biofluids without prior enzyme treatment, 114 were known as food components (out of the 502 known food polyphenols in Phenol‐Explorer) (Fig. 1D). The latter correspond to compounds absorbed in the body without further biotransformation. The remaining 187 metabolites correspond to methylated derivatives and other metabolites formed in host tissues or by microbiota.

**Figure 2:**
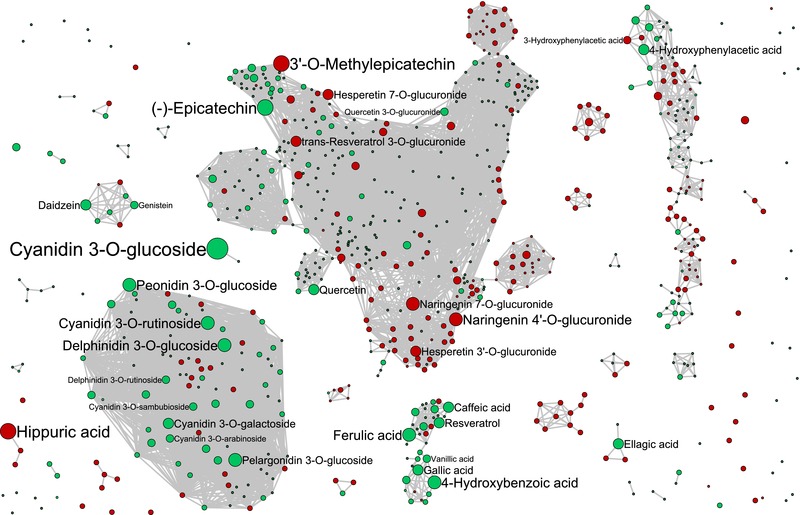
Chemical similarity map of the polyphenol metabolome showing the names of the most frequently detected polyphenol metabolites from 221 publications. Node and label size are proportional to the number of intervention studies in which each metabolite has been reported in biofluids. Similar compounds (Tanimoto chemical similarity >70%) are linked by gray edges. Red nodes correspond to metabolites that have been detected only in biofluids. Green nodes correspond to metabolites that have been detected in biofluids and are also food components.

Mapping of the polyphenol metabolome by chemical similarity revealed distinct clusters of related metabolites (Fig. 2). The largest cluster, in the central part of the chemical similarity map, contains glucuronides of all polyphenol classes and subclasses, as well as some glycosides. The anthocyanins (mono‐ and diglycosides) are chemically distinct from all other metabolites and account for the next largest cluster. Hydroxycinnamic acids and benzoic acids are seen clustered at the bottom and middle and left. Compounds found only in biofluids and those also known as food components are indicated by red and green nodes, respectively. The former includes glucuronides, sulfate esters, *O*‐methylated compounds (e.g. 3′‐*O*‐methylepicatechin, and *O*‐methylated anthocyanins in the center of the anthocyanin cluster) and microbial metabolites (such as dihydrogenated isoflavones and hydroxycinnamic acids, hippuric acid, and urolithin C). The most commonly reported metabolites are emphasized with nodes sizes proportional to the number of interventions in which they were identified. See Supporting Information 3 for full metabolite labels.

### 3.3 Pharmacokinetics

**Figure 3:**
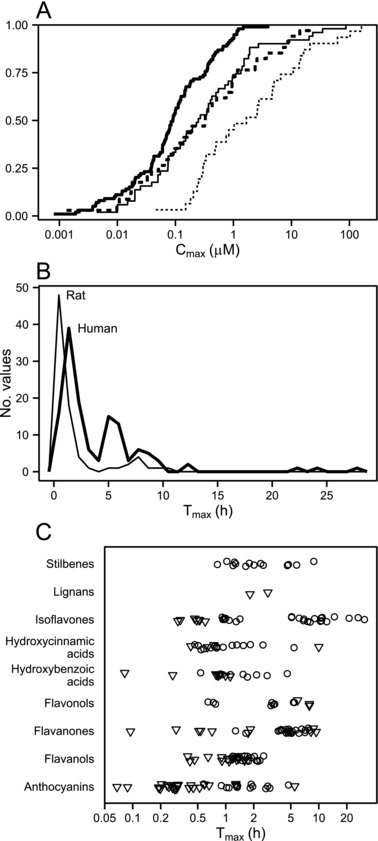
Summary of polyphenol pharmacokinetics. (A) Empirical cumulative density function showing distribution of *C*_max_ values collected. Bold and normal lines represent human and rat data, respectively; solid and dashed lines represent foods (including experimental foods) and pure compound doses, respectively. (B) Frequency polygon of *T*_max_ values. Bold and normal lines represent human and rat data, respectively. (C) Variation of *T*_max_ with polyphenol subclass and species (circles, human; triangles, rat).

Of the 383 metabolites in Phenol‐Explorer, 235 were quantified in biofluids at multiple time points and pharmacokinetics described. A total of 222 maximum plasma concentration (*C*_max_) values were collected for 88 metabolites. In humans, a median *C*_max_ of 0.1 μM (interquartile range = 0.37 μM) was observed after food or dietary supplement consumption. Stratification of this distribution by dose type revealed a shifted distribution of values for pure compounds compared to foods (median values of 0.32 and 0.09 μM, respectively; Fig. 3A). In rats, where pure polyphenols were more often administered, higher maximum concentrations were achieved (median = 0.41 μM, interquartile range = 2.25 μM). In relation to time taken to reach maximum plasma concentration (*T*_max_), 207 values were collected corresponding to 81 metabolites. Median *T*_max_ was much shorter in rat than in human (median time = 0.71 versus 2.18 h). In rat, peak concentrations usually occurred within 1 h of polyphenol administration and few values >5 h were observed (Fig. 3B). In humans, *T*_max_ of flavanone and isoflavone metabolites was usually in excess of 5 h (Fig. 3C). For most other subclasses, *T*_max_ was usually less than 2 h. Seventy‐one plasma half‐life values were compiled for the Phenol‐Explorer database. Median half‐life for all polyphenol metabolites in humans was 2.8 h (interquartile range = 4.05 h).

The proportion of dose excreted in urine was measured for 80 compounds until 24 h or more after administration. For extracts incubated with either β‐glucuronidase or sulfatase to group all conjugates with a common parent polyphenol, proportion of dose recovered could be used as a measure of overall bioavailability. Median urinary excretion was 10.9%, although this varied substantially between different polyphenols (interquartile range = 25.9%) (Supporting Information 4). Some were particularly poorly recovered (e.g. 0.01% for some anthocyanins) either due to poor absorption in the gut or to extensive biotransformation. The highest recoveries in humans were observed for stilbenes, tyrosols, and isoflavonoids (20–60%).

### 3.4 Frequently reported metabolites from polyphenol‐rich and pure polyphenol doses

The most frequently identified metabolites (without enzyme treatment of biofluids) in Phenol‐Explorer were determined based on the number of intervention studies in which each was described (Table 2). For many of these, food sources are available in Phenol‐Explorer as well as the identities of precursors administered as pure compounds. The most commonly reported metabolite was cyanidin 3‐*O*‐glucoside, which appeared in plasma in 40 different interventions after administration of different berries, berry extracts, and pure cyanidin 3‐*O*‐glucoside. Delphinidin and peonidin glucosides were also commonly reported in these interventions. From the flavanol subclass, epicatechin and 3‐*O*‐methylepicatechin were frequently observed in biofluids, usually after experimental doses of cocoa and tea. The most frequently observed glucuronides were those of hesperetin and naringenin, usually originating from citrus fruits. The phenolic acids 4‐hydroxybenzoic acid, 4‐hydroxyphenylacetic acid, gallic acid, and the most downstream polyphenol metabolite, hippuric acid, were frequently identified as metabolites of a diverse range of polyphenols.

**Table 2:** The 25 most commonly identified polyphenol metabolites in human and animal biofluids across 424 intervention studies from 221 publications

Polyphenol metabolite (polyphenol subclass)	Number of interventions in which detected (number of experimental sources)^a^	Main food doses	Pure compound doses	Highest *C*_max_ (μM)^b^^,^ c		*T*_max_ value or range (h)^b),c)^	
				Human	Rat	Human	Rat
Cyanidin 3‐*O*‐glucoside (anthocyanin)	40 (34)	Various berries, berry juices and extracts	Cyanidin 3*‐O*‐glucoside	0.04	0.3	1.09	0.25
Hippuric acid (nonphenolic metabolite)	23 (19)	Cranberry powder, green tea and green tea solids, wheat bran, poplar wood lignins, orange juice, blackcurrant and raspberry juices	Catechin, ferulic acid, sinapic acid	—	—	—	—
(−)‐Epicatechin (flavanol)	16 (7)	Green tea, cocoa powder	(−)‐Epicatechin, procyanidin B2	—	0.25	—	0.5
Resveratrol, *cis* or *trans* (stilbene)	16 (3)	Red wine, *Polygonum cuspidatum* extract	Resveratrol	2.36		0.86–1.50	—
3′‐*O*‐Methylepicatechin (flavanol)	15 (5)	Cocoa powder	(−)‐Epicatechin, procyanidin B2	—	0.55	—	0.5–1.5
4‐Hydroxybenzoic acid (hydroxybenzoic acid)	15 (12)	Green tea, cranberry powder, raspberry and orange juices, virgin olive oil, blueberry	Pelargonidin	—	—	—	—
Delphinidin 3‐*O*‐glucoside (anthocyanin)	15 (14)	Berries and berry extracts, blood orange juice	Delphinidin 3‐*O*‐glucoside	—	0.15	—	0.25–5.00
Cyanidin 3‐*O*‐rutinoside (anthocyanin)	14 (14)	Berries and berry extracts, blackcurrant and raspberry juices	—	0.04	0.23	1.64	0.25–1.00
Ferulic acid (hydroxycinnamic acid)	14 (11)	Perilla extract, cranberry powder, green coffee extract, cranberry and raspberry juices	Caffeic acid, dihydrocaffeic acid, ferulic acid	0.12–0.36	1.68	0.5–1.0	0.5
Peonidin 3‐*O*‐glucoside (anthocyanin)	14 (13)	Berries and berry extracts, cranberry and raspberry juices	Cyanidin 3‐*O*‐glucoside	0.0008	1.4	0.05	0.25
4‐Hydroxyphenylacetic acid (hydroxyphenylacetic acid)	12 (9)	Cranberry powder, cranberry, orange and raspberry juices, green tea		—	—	—	—
Daidzein (isoflavone)	12 (11)	Soy protein, soy beverages, soy milk and supplements	Daidzein	2.54	—	1.0–9.1	—
Pelargonidin 3‐*O*‐glucoside (anthocyanin)	12 (6)	Strawberry, marionberry powder	—	—	—	—	—
Naringenin 7‐*O*‐glucuronide (flavanone)	11 (8)	Orange and grapefruit juices, orange	Naringenin	0.77	—	1.63–6.40	—
Cyanidin 3‐*O*‐galactoside (anthocyanin)	10 (10)	Blueberry, lingonberry, cranberry juice, other berry extracts	—	0.02	0.19	2.5	0.25
Gallic acid (hydroxybenzoic acid)	10 (8)	Cranberry powder, Shuangdan granules, green tea, blueberry	Ethyl gallate	—	1.03	—	0.2
Hesperetin 7‐*O*‐glucuronide (flavanone)	10 (7)	Orange, orange juice, polyphenol‐rich drink	Hesperidin	1.48	—	4.6–7.3	—
Naringenin 4′‐*O*‐glucuronide (flavanone)	10 (7)	Orange and grapefruit juices, orange	—	0.77	—	1.63–6.40	—
Quercetin (flavonol)	10 (9)	White wine, ginkgo biloba tablet, blackcurrant juice, yellow and red onions, shallots	Quercetin, quercetin 3‐*O*‐glucoside, quercetin 4′‐*O*‐glucoside	0.05, 3.95	—	2.33–3.60	—
3‐Hydroxyphenylacetic acid	9 (7)	Cranberry powder, orange juice fortified with hesperetin, cranberry juice fortified with rutin	—	—	—	—	
Caffeic acid (hydroxycinnamic acid)	9 (6)	Cranberry powder, shuangdan granules	Caffeic acid, eriocitrin	0.09	—	1.5–3.0	—
Genistein	9 (8)	Soy protein, soy milk, soy protein beverage, soy supplements		1.02	0.48	1.0–5.9	0.5
Ellagic acid (hydroxybenzoic acid)	9 (9)	Pomegranate extract, raspberry	Punicalagin	0.11	—	0.65–2.58	—
Resveratrol 3‐*O*‐glucuronide, cis or trans) (stilbene)	8 (8)	Red, white, and sparkling wine	Piceid	0.16	—	6	—
Vanillic acid (hydroxybenzoic acid)	8 (6)	Cranberry powder, blackcurrant juice, cocoa powder, virgin olive oil	—	—	—	—	—

a Only interventions that did not enzymatically hydrolyze biofluids are counted.

b Pharmacokinetic data for aglycones represent all corresponding conjugates following enzymatic hydrolysis.

c Pharmacokinetic data are omitted where metabolites were derived from several different precursors.

**Figure 4:**
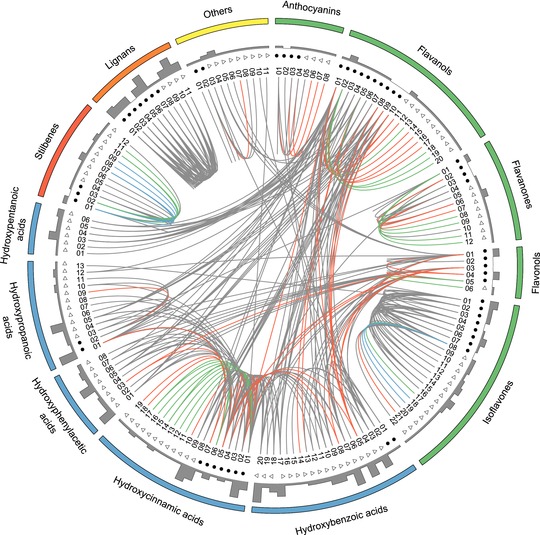
Circular diagram of polyphenol metabolic pathways derived from studies in which pure compounds were orally administered to humans and animals. Compounds are ordered by subclass and numbered. Filled circles represent pure compounds administered in intervention studies and empty triangles represent metabolites found in urine or plasma. Histograms indicate the numbers of precursors leading to the formation of each metabolite (scale 0–8 precursors). Link color represents metabolism reactions as follows: red, methylation; green, glucuronidation; blue, sulfation; gray, combination of reactions or unchanged from precursor. See Supporting Information 5 for compound codes.

Administration of 59 pure polyphenol doses, mostly to rats, led to the identification of 160 different metabolites in biofluids. Doses of 5‐caffeoylquinic acid, which is particularly well studied as a major coffee phenol, led to the identification of 27 circulating metabolites across all studies, while caffeic acid and epicatechin were each the precursor of 22 derivatives. Over half of all metabolites identified in biofluids in interventions with pure polyphenols were, however, derived from a single precursor only. The metabolism of these pure polyphenol doses is represented in Fig. 4.

## 4 Discussion

Biological databases are essential for the efficient retrieval of relevant information from large numbers of scientific publications 17. In the present study, all polyphenol metabolites reliably identified in human and animal biofluids and described in literature were collated and regarded as the experimentally determined polyphenol metabolome. This is only a small subset of all metabolites theoretically possible, since coverage is governed by research design and analytical capabilities. For example, less glucuronides and sulfates and more aglycones and esters were described in intervention studies included in Phenol‐Explorer than might be expected. Conjugates are indeed difficult to identify and the metabolites whose position of conjugation were unknown were not included in Phenol‐Explorer. Also, a striking number of unchanged glycosides were found in biofluids; most were anthocyanins, recovered in low concentrations, which escaped deglycosylation by luminal enzymes and can be transported into cells by bilitranslocase membrane proteins 18, 19. In addition, several glycosides of other polyphenol subclasses were either detected and quantified (hesperidin, naringin, neohesperidin, puerarin, and quercetin 3‐*O*‐rutinoside) or detected only (6′′‐*O*‐malonyldaidzin, daidzin, glycitin, isorhamnetin 3‐*O*‐glucoside, quercetin 3,4′‐*O*‐diglucoside, quercetin 3,4′‐*O*‐diglucoside, and quercetin 3‐*O*‐glucoside) in biofluids of at least one volunteer or animal study.

Absorption and pharmacokinetics govern the extent to which polyphenols reach target tissues via systemic circulation. Phenol‐Explorer data show that plasma concentrations > 10 μM are possible but implausible from human diets, and typical plasma concentrations of individual polyphenol metabolites are many times lower (Fig. 3A). The greatest C_max_ recorded in humans from an unmodified food was 3.95 μM quercetin from a dose of shallot skin 20, although the consumption of this tissue alone is improbable. The highest C_max_ recorded for a polyphenol following a dose of any commonly‐consumed food tested was 1.21 μM dihydroferulic acid following intake of instant coffee (4 g instant coffee powder in hot water) 21. In both cases, glucuronides and sulfate esters were hydrolyzed before analysis. However, overall high levels of polyphenol exposure may be experienced upon the simultaneous consumption of different polyphenol‐rich foods containing many different polyphenols. Upon ingestion of such a meal, total polyphenol plasma concentrations in excess of 5 μM might be possible. Time taken to reach these peak concentrations is also important, since metabolites which are absorbed gradually and persist in circulation may be more likely to reach target tissues. T_max_ values collected for Phenol‐Explorer clustered at around 2 h and 5 h after polyphenol doses in humans, corresponding to absorption in the small and large intestine, respectively. In rat models, small intestine absorption was quicker than in humans and late T_max_ was less frequently observed. Care should be taken when extrapolating pharmacokinetic data from rat models to human metabolism, particularly as metabolizing enzymes show different expression profiles 22.

Some polyphenol metabolites were identified in many different interventions. Cyanidin 3‐glucoside was detected in the most, identified in 40 separate intervention studies following ingestion of many different berries and berry extracts. However, the flavanol aglycones epicatechin and 3‐methylepicatechin were detected after administration of a greater range of polyphenol‐rich foodstuffs. Fewer phase II conjugates were reported in multiple interventions because their characterization requires greater analytical precision. Those most often reported were different isoforms of naringenin and hesperetin glucuronides, which originate from flavanone glycosides in citrus fruits. Other metabolites were notable for being derived from a wide range of polyphenols. The ring‐cleavage microbial metabolite hippuric acid was identified in biofluids after the intake of sources of polyphenols belonging to all most important subclasses (Fig. 4, compound #20). Previous studies have shown the compound to increase upon total polyphenol and fruit intake 23, 24 and thus excretion has been postulated to reflect polyphenol intake. It may also however be derived from aromatic amino acids 25 and thus is usually excreted in urine at low levels, independently of phenol intake. In the present study, hippuric acid was often associated with its closest precursor 4‐hydroxybenzoic acid (Fig. 4, hydroxybenzoic acids, compound #07), which itself was one of the most commonly reported metabolites in the database. This compound, like 3‐ and 4‐hydroxyphenylacetic acids, is a product of microbial polyphenol metabolism 26, 27 and could account for substantial proportions of the ingested polyphenol doses. This is illustrated by the many pathways associating more particularly parent hydroxycinnamates, flavanols, and flavonols with various phenolic acid metabolites shown in Fig. 4 (hydroxypentanoic acids, hydroxypropanoic acids, hydroxyphenylacetic acids, and hydroxybenzoic acids). In particular, many of these microbial metabolites are formed from various phenolic precursors (see Fig. 4, grey histograms).

There is much interest in the discovery of biomarkers of polyphenol‐rich food intake or of total or individual polyphenol intake, since reliable estimates of dietary exposure are necessary to better understand the health effects of polyphenols through epidemiological studies. Biomarkers could measure polyphenol intake more objectively than dietary questionnaires, which are susceptible to different types of bias 28. Detailed information on metabolism and pharmacokinetics, such as is available in Phenol‐Explorer, is useful as a starting point in biomarker discovery but also aids the identification of metabolites, detected in biofluids, which discriminate high and low consumers of a food of interest. Phenol‐Explorer data, e.g., support the validity of biomarkers, such as naringenin and hesperetin glucuronides, recently proven to reflect citrus fruit intake reliably 13. Biomarkers of exposure will be essential for establishing relationships between polyphenol intake and disease risk, particularly for diseases such as cancer where links are currently poorly characterized 5.

The present study is the first to describe and visualize the polyphenol metabolome as currently known. However, certain limitations should be kept in mind. First, despite a much deeper understanding of metabolism than only a few years ago, only a small proportion of the possible polyphenol metabolome has been described. In general, studies have disproportionately concentrated on the principal flavonoid and phenolic acid subclasses, and more studies on lignans and complex polyphenols, such as proanthocyanidin and theaflavin polymers are needed. A lack of detailed knowledge is evident for some commonly consumed subclasses, such as anthocyanins, which are unstable and quickly break down to smaller products 29. As enzymatic hydrolyses are often employed, relatively few phase II conjugates have been confirmed, and these have great potential as specific biomarkers of intake of individual polyphenols and polyphenol‐containing foods. There is evidence that over 500 polyphenols are known to be consumed by humans, but in our analysis the administration of only 59 pure polyphenols to humans or rats led to the identification of 160 polyphenol metabolites. Second, more studies administering pure compounds are required to precisely identify metabolites of interest. A particular limitation of the present analysis is the proportion of pure polyphenol studies performed on animals (98 experimental interventions) compared to humans (32 interventions). Thus knowledge obtained from pure polyphenol doses is derived predominantly from animals. Despite these drawbacks, the knowledge of the polyphenol metabolome summarized here is needed both to understand in vivo bioactivity and to aid in the search for biomarkers for application to the study of polyphenol intake in relation to health.

*The authors have declared no conflict of interest*.

## Supporting information

As a service to our authors and readers, this journal provides supporting information supplied by the authors. Such materials are peer reviewed and may be re‐organized for online delivery, but are not copy‐edited or typeset. Technical support issues arising from supporting information (other than missing files) should be addressed to the authors.

Supplementary Material

## References

[1] Perez‐Jimenez, J. , Neveu, V. , Vos, F. , Scalbert, A. , Systematic analysis of the content of 502 polyphenols in 452 foods and beverages: an application of the phenol‐explorer database. J. Agric. Food Chem. 2010, 58, 4959–4969. 20302342 10.1021/jf100128b

[2] Perez‐Jimenez, J. , Fezeu, L. , Touvier, M. , Arnault, N. et al., Dietary intake of 337 polyphenols in French adults. Am. J. Clin. Nutr. 2011, 93, 1220–1228. 21490142 10.3945/ajcn.110.007096

[3] Arts, I. C. , Hollman, P. C. , Polyphenols and disease risk in epidemiologic studies. Am. J. Clin. Nutr. 2005, 81, 317S–325. 15640497 10.1093/ajcn/81.1.317S

[4] Scalbert, A. , Manach, C. , Morand, C. , Rémésy, C. et al., Dietary polyphenols and the prevention of diseases. Crit. Rev. Food Sci. Nutr. 2005, 45, 287–306. 16047496 10.1080/1040869059096

[5] Zamora‐Ros, R. , Touillaud, M. , Rothwell, J. A. , Romieu, I. et al., Measuring exposure to the polyphenol metabolome in observational epidemiologic studies: current tools and applications and their limits. Am. J. Clin. Nutr. 2014, 100, 11–26. 24787490 10.3945/ajcn.113.077743 PMC4144095

[6] Neveu, V. , Perez‐Jimenez, J. , Vos, F. , Crespy, V. et al., Phenol‐Explorer: an online comprehensive database on polyphenol contents in foods. Database 2010, 2010, bap024. 20428313 10.1093/database/bap024 PMC2860900

[7] Rothwell, J. A. , Urpi‐Sarda, M. , Boto‐Ordonez, M. , Knox, C. et al., Phenol‐Explorer 2.0: a major update of the Phenol‐Explorer database integrating data on polyphenol metabolism and pharmacokinetics in humans and experimental animals. Database 2012, 2012, bas031. 22879444 10.1093/database/bas031 PMC3414821

[8] Manach, C. , Williamson, G. , Morand, C. , Scalbert, A. et al., Bioavailability and bioefficacy of polyphenols in humans. I. Review of 97 bioavailability studies. Am. J. Clin. Nutr. 2005, 81, 230S‐242S. 15640486 10.1093/ajcn/81.1.230S

[9] Perez‐Jimenez, J. , Hubert, J. , Hooper, L. , Cassidy, A. et al., Urinary metabolites as biomarkers of polyphenol intake in humans: a systematic review. Am. J. Clin. Nutr. 2010, 92, 801–809. 20810980 10.3945/ajcn.2010.29924

[10] Quiñones, M. , Miguel, M. , Aleixandre, A. , Beneficial effects of polyphenols on cardiovascular disease. Pharmacol. Res. 2013, 68, 125–131. 23174266 10.1016/j.phrs.2012.10.018

[11] Ramos, S. , Cancer chemoprevention and chemotherapy: Dietary polyphenols and signalling pathways. Mol. Nutr. Food Res. 2008, 52, 507–526. 18435439 10.1002/mnfr.200700326

[12] Boto‐Ordonez, M. , Rothwell, J. , Andres‐Lacueva, C. , Manach, C. et al., Prediction of the metabolic profile of polyphenols after intake of wine and wine products: an application of the Phenol‐Explorer database. Mol. Nutr. Food Res. 2014, 58, 466–477. 24123832 10.1002/mnfr.201300411

[13] Pujos‐Guillot, E. , Hubert, J. , Martin, J. F. , Lyan, B. et al., Mass spectrometry‐based metabolomics for the discovery of biomarkers of fruit and vegetable intake: citrus fruit as a case study. J. Proteome Res. 2013, 12, 1645–1659. 23425595 10.1021/pr300997c

[14] Barupal, D. K. , Haldiya, P. K. , Wohlgemuth, G. , Kind, T. et al., MetaMapp: mapping and visualizing metabolomic data by integrating information from biochemical pathways and chemical and mass spectral similarity. BMC Bioinformatics 2012, 13, 99. 22591066 10.1186/1471-2105-13-99 PMC3495401

[15] Smoot, M. E. , Ono, K. , Ruscheinski, J. , Wang, P.‐L. et al., Cytoscape 2.8: new features for data integration and network visualization. Bioinformatics 2011, 27. 10.1093/bioinformatics/btq675 PMC3031041 21149340

[16] Krzywinski, M. I. , Schein, J. E. , Birol, I. , Connors, J. et al., Circos: an information aesthetic for comparative genomics. Genome Res. 2009, 19, 1639–1645. 19541911 10.1101/gr.092759.109 PMC2752132

[17] Scalbert, A. , Andres‐Lacueva, C. , Arita, M. , Kroon, P. et al., Databases on food phytochemicals and their health‐promoting effects. J. Agric. Food Chem. 2011, 59, 4331–4348. 21438636 10.1021/jf200591d

[18] Prior, R. L. , Wu, X. L. , Anthocyanins: structural characteristics that result in unique metabolic patterns and biological activities. Free Radic. Res. 2006, 40, 1014–1028. 17015246 10.1080/10715760600758522

[19] Passamonti, S. , Terdoslavich, M. , Franca, R. , Vanzo, A. et al., Bioavailability of flavonoids: a review of their membrane transport and the function of bilitranslocase in animal and plant organisms. Curr. Drug Metab. 2009, 10, 369–394. 19519345 10.2174/138920009788498950

[20] Wiczkowski, W. , Romaszko, J. , Bucinski, A. , Szawara‐Nowak, D. et al., Quercetin from shallots (Allium cepa L. var. aggregatum) is more bioavailable than its glucosides. J. Nutr. 2008, 138, 885–888. 18424596 10.1093/jn/138.5.885

[21] Guy, P. A. , Renouf, M. , Barron, D. , Cavin, C. et al., Quantitative analysis of plasma caffeic and ferulic acid equivalents by liquid chromatography tandem mass spectrometry. J. Chromatogr. B Analyt. Technol. Biomed. Life Sci. 2009, 877, 3965–3974. 10.1016/j.jchromb.2009.10.006 19879819

[22] Cao, X. , Gibbs, S. T. , Fang, L. , Miller, H. A. et al., Why is it challenging to predict intestinal drug absorption and oral bioavailability in human using rat model. Pharm. Res. 2006, 23, 1675–1686. 16841194 10.1007/s11095-006-9041-2

[23] DuPont, M. S. , Bennett, R. N. , Mellon, F. A. , Williamson, G. , Polyphenols from alcoholic apple cider are absorbed, metabolized and excreted by humans. J. Nutr. 2002, 132, 172–175. 11823574 10.1093/jn/132.2.172

[24] Toromanovic, J. , Kovac‐Besovic, E. , Sapcanin, A. , Tahirovic, I. et al., Urinary hippuric acid after ingestion of edible fruits. Bosn. J. Basic Med. Sci. 2008, 8, 38–43. 18318670 10.17305/bjbms.2008.2994 PMC5724873

[25] Rechner, A. R. , Spencer, J. P. , Kuhnle, G. , Hahn, U. et al., Novel biomarkers of the metabolism of caffeic acid derivatives in vivo. Free Radic. Biol. Med. 2001, 30, 1213–1222. 11368919 10.1016/s0891-5849(01)00506-8

[26] Aura, A. M. , O'Leary, K. A. , Williamson, G. , Ojala, M. et al., Quercetin derivatives are deconjugated and converted to hydroxyphenylacetic acids but not methylated by human fecal flora in vitro. J. Agric. Food Chem. 2002, 50, 1725–1730. 11879065 10.1021/jf0108056

[27] Aura, A. M. , Martin‐Lopez, P. , O'Leary, K. A. , Williamson, G. et al., In vitro metabolism of anthocyanins by human gut microflora. Eur. J. Nutr. 2005, 44, 133–142. 15309431 10.1007/s00394-004-0502-2

[28] Kristal, A. R. , Peters, U. , Potter, J. D. , Is It Time to Abandon the Food Frequency Questionnaire? Cancer Epidemiol. Biomarkers Prev. 2005, 14, 2826–2828. 16364996 10.1158/1055-9965.EPI-12-ED1

[29] Czank, C. , Cassidy, A. , Zhang, Q. , Morrison, D. J. et al., Human metabolism and elimination of the anthocyanin, cyanidin‐3‐glucoside: a 13C‐tracer study. Am. J. Clin. Nutr. 2013, 97, 995–1003. 23604435 10.3945/ajcn.112.049247

