# Supplementary material for: Systematic analysis of the polyphenol metabolome using the Phenol‐Explorer database
Source: Mol Nutr Food Res. 2015 Oct 15;60(1):203–11. doi: 10.1002/mnfr.201500435 (PMC5057353; doi:10.1002/mnfr.201500435)
Supplement: Supplementary file 1 — Supplementary Material [file MNFR-60-203-s001.docx]

## Online supplemental material

**Supplemental data 1.** Classification of polyphenols as described in the Phenol-Explorer database.

| Class | | Subclass | |
| --- | --- | --- | --- |
| Flavonoids | | Anthocyanins, chalcones, dihydrochalcones, dihydroflavonols, flavanols, flavanones, flavones, flavonols, isoflavonoids | |
| Phenolic acids | | Hydroxybenzoic acids, hydroxycinnamic acids, hydroxyphenylacetic acids, hydroxyphenyllactic acids, hydroxyphenylpropanoic acids, hydroxyphenylpentanoic acids | |
| Lignans | |  | |
| Stilbenes | |  | |
| Other phenols | | Alkylmethoxyphenols, alkylphenols, betacyanins, capsaicinoids, curcuminoids, dihydrocapsaicins, furanocoumarins, hydroxybenzaldehydes, hydroxybenzoketones, hydroxycinnamaldehydes, hydroxycoumarins, hydroxyphenylalcohols, hydroxyphenylpropenes, methoxyphenols, naphtoquinones, phenolic terpenes, tyrosols | |

**Supplemental data 2**. Types of polyphenol metabolite stored in Phenol-Explorer, by polyphenol class.


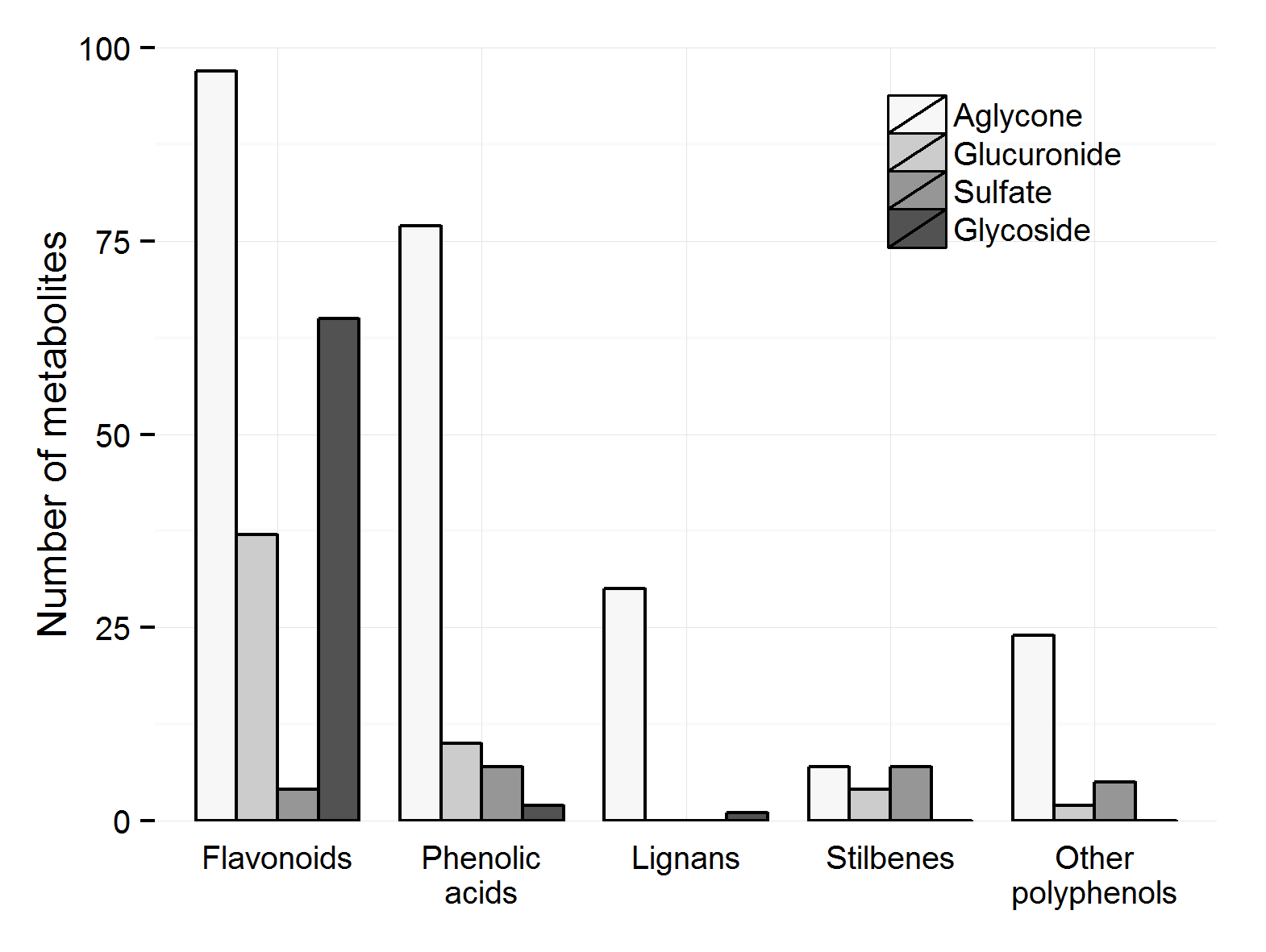


**Supplemental data 3**. Chemical similarity map as shown in manuscript Figure 2 with all metabolite labels.

**Supplemental data 3**. Percentage dose of polyphenol recovered from urine, by polyphenol subclass. Triangles and circles represent rat and human data respectively.


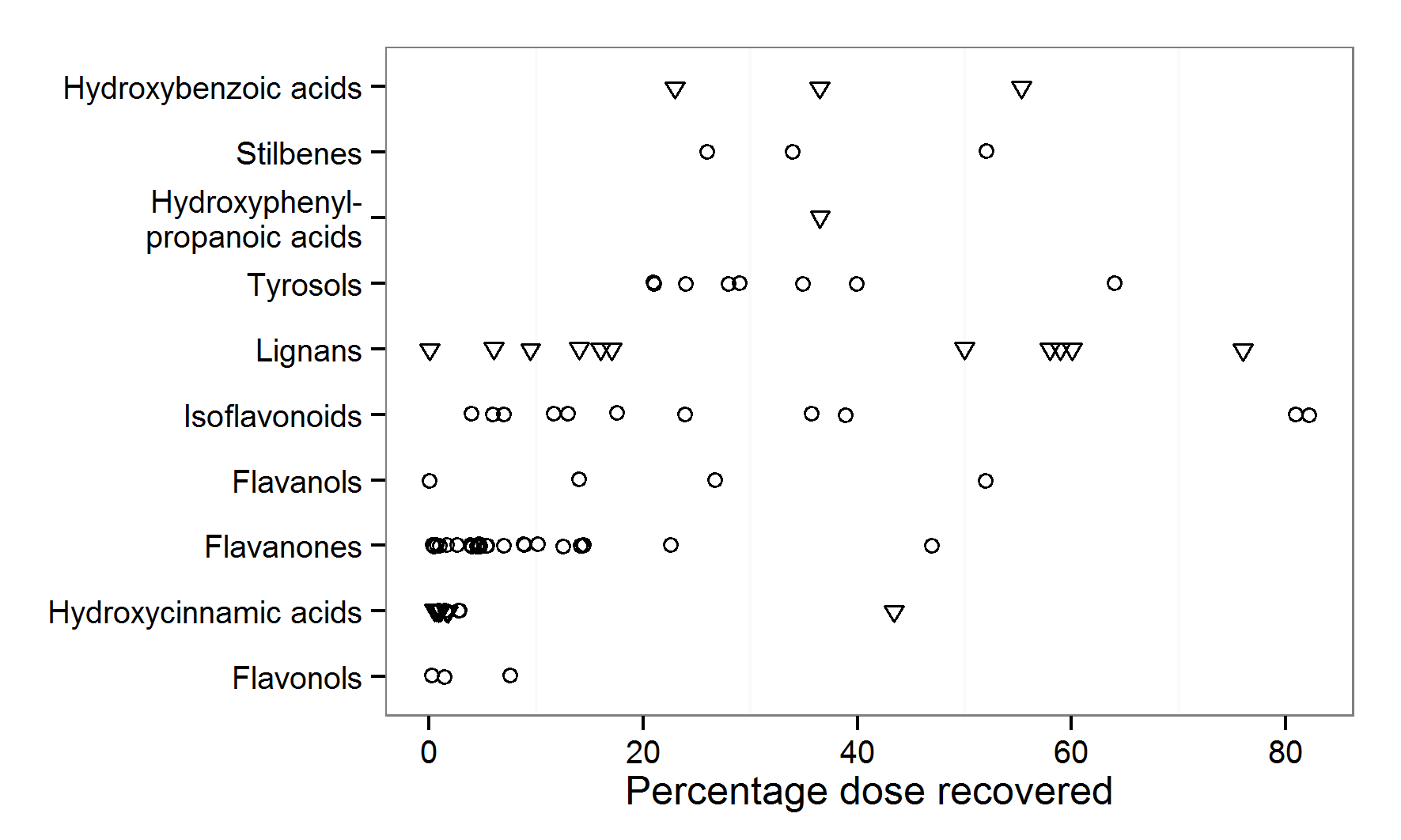


**Supplemental data 4**. Key to compound codes in manuscript Figure 4.

| Polyphenol | | Compound number | |
| --- | --- | --- | --- |
| **Anthocyanins**  Cyanidin 3-O-glucoside | | 01 | |
| Pelargonidin | | 02 | |
| Delphinidin 3-O-glucoside | | 03 | |
| Delphinidin 3-O-rutinoside | | 04 | |
| Peonidin 3-O-glucoside | | 05 | |
| 4'-O-Methylcyanidin 3-O-D-glucoside | | 06 | |
| 4-O-Methyldelphinidin 3-O-D-glucoside | | 07 | |
| 4'-O-Methyldelphinidin 3-O-rutinoside | | 08 | |
| **Flavanols**  Catechin | | 01 | |
| Epicatechin | | 02 | |
| Epigallocatechin | | 03 | |
| Epicatechin 3-O-gallate | | 04 | |
| Epigallocatechin 3-O-gallate | | 05 | |
| Theaflavin | | 06 | |
| Procyanidin dimer B2 | | 07 | |
| Procyanidin dimer B3 | | 08 | |
| Procyanidin trimer C2 | | 09 | |
| Procyanidins, total | | 10 | |
| 3'-O-Methylepicatechin | | 11 | |
| 4'-O-Methyl-(-)-epicatechin 3'-O-glucuronide | | 12 | |
| Epicatechin 3'-O-glucuronide | | 13 | |
| 3'-O-Methylcatechin | | 14 | |
| 4',4"-O-Dimethylepigallocatechin 3-O-gallate | | 15 | |
| 4''-O-Methylepigallocatechin 3-O-gallate | | 16 | |
| 4'-O-Methylepicatechin | | 17 | |
| Epigallocatechin 3-O-gallate-7-O-glucoside-4''-O-glucuronide | | 18 | |
| 3'-O-Methyl-(-)-epicatechin 7-O-glucuronide | | 19 | |
| Epicatechin 7-O-glucuronide | | 20 | |
| **Flavanones**  Naringenin | | 01 | |
| Eriocitrin | | 02 | |
| Hesperidin | | 03 | |
| Naringin | | 04 | |
| Eriodictyol | | 05 | |
| Hesperetin | | 06 | |
| Isosakuranetin | | 07 | |
| Hesperetin 3'-O-glucuronide | | 08 | |
| Hesperetin 7-O-glucuronide | | 09 | |
| Homoeriodictyol | | 10 | |
| Naringenin 5-O-glucuronide | | 11 | |
| Naringenin 7-O-glucuronide | | 12 | |
| **Flavonols**  Quercetin | | 01 | |
| Quercetin 3-O-glucoside | | 02 | |
| Quercetin 3-O-rutinoside | | 03 | |
| Isorhamnetin | | 04 | |
| Quercetin 4'-O-glucoside | | 05 | |
| Quercetin 3-O-glucuronide | | 06 | |
| **Isoflavones**  Daidzein | | 01 | |
| Genistein | | 02 | |
| Biochanin A | | 03 | |
| Daidzin | | 04 | |
| Puerarin | | 05 | |
| Tectorigenin | | 06 | |
| Tectoridin | | 07 | |
| Kakkalide | | 08 | |
| 3'-Hydroxydaidzein | | 09 | |
| 6,7,4'-Trihydroxyisoflavone | | 10 | |
| 6'-Hydroxy-O-desmethylangolensin | | 11 | |
| 7,8,4'-Trihydroxyisoflavone | | 12 | |
| Dihydrodaidzein | | 13 | |
| Dihydrogenistein | | 14 | |
| Equol | | 15 | |
| O-Desmethylangolensin | | 16 | |
| Irisolidone 7-O-glucuronide | | 17 | |
| Tectorigenin 7-sulfate | | 18 | |
| Tectorigenin 4'-sulfate | | 19 | |
| Irisolidone | | 20 | |
| 5,7-Dihydroxy-8,4'-dimethoxyisoflavone | | 21 | |
| Isotectorigenin | | 22 | |
| **Hydroxybenzoic acids**  Gallic acid ethyl ester | | 01 | |
| Punicalagin | | 02 | |
| Protocatechuic acid | | 03 | |
| Gallic acid | | 04 | |
| Vanillic acid | | 05 | |
| Ellagic acid | | 06 | |
| 4-Hydroxybenzoic acid | | 07 | |
| Syringic acid | | 08 | |
| Benzoic acid | | 09 | |
| 3-Hydroxybenzoic acid | | 10 | |
| 2,4-Dihydroxybenzoic acid | | 11 | |
| 3,5-Dihydroxybenzoic acid | | 12 | |
| 3-O-Methylgallic acid | | 13 | |
| 4-O-Methylgallic acid | | 14 | |
| Gallagic acid | | 15 | |
| Punicalin | | 16 | |
| 4-Hydroxyhippuric acid | | 17 | |
| 3-Hydroxyhippuric acid | | 18 | |
| 2-Hydroxyhippuric acid | | 19 | |
| Hippuric acid | | 20 | |
| **Hydroxycinnamic acids**  Caffeic acid | | 01 | |
| Ferulic acid | | 02 | |
| Rosmarinic acid | | 03 | |
| Sinapic acid | | 04 | |
| 5-Caffeoylquinic acid | | 05 | |
| Isoferulic acid | | 06 | |
| 1,5-Dicaffeoylquinic acid | | 07 | |
| p-Coumaric acid | | 08 | |
| m-Coumaric acid | | 09 | |
| 3-O-Methylrosmarinic acid | | 10 | |
| Isoferulic acid 3-O-glucuronide | | 11 | |
| Ferulic acid 4-O-glucuronide | | 12 | |
| Feruloyl C1-glucuronide | | 13 | |
| Isoferuloyl C1-glucuronide | | 14 | |
| Caffeic acid 3-O-glucuronide | | 15 | |
| Caffeic acid 4-O-glucuronide | | 16 | |
| Caffeoyl C1-glucuronide | | 17 | |
| 1-Caffeoyl-5-feruloylquinic acid | | 18 | |
| 1-Feruloyl-5-caffeoylquinic acid | | 19 | |
| **Hydroxyphenylacetic acids**  3,4-Dihydroxyphenylacetic acid | | 01 | |
| 4-Hydroxyphenylacetic acid | | 02 | |
| Homovanillic acid | | 03 | |
| Methoxyphenylacetic acid | | 04 | |
| 3-Hydroxyphenylacetic acid | | 05 | |
| 2-Hydroxyphenylacetic acid | | 06 | |
| Phenylacetic acid | | 07 | |
| 2-Hydroxy-2-phenylacetic acid | | 08 | |
| **Hydroxypropanoic acids**  Dihydrocaffeic acid | | 01 | |
| Danshensu | | 02 | |
| Dihydro-p-coumaric acid | | 03 | |
| 3-(3,4-Dihydroxyphenyl)-2-methoxypropionic acid | | 04 | |
| 3-Hydroxyphenylpropionic acid | | 05 | |
| Dihydroferulic acid | | 06 | |
| Dihydrosinapic acid | | 07 | |
| Dihydroferuloylglycine | | 08 | |
| 3-Methoxy-4-hydroxyphenyllactic acid | | 09 | |
| 3,4-Dihydroxyphenyllactic acid methyl ester | | 10 | |
| Hydroxydanshensu | | 11 | |
| 3-Phenylpropionic acid | | 12 | |
| 4-Hydroxyphenyl-2-propionic acid | | 13 | |
| **Hydroxypentanoic acids**  5-(3'-Methoxy-4'-hydroxyphenyl)-γ-valerolactone | | 01 | |
| 5-(3',4'-dihydroxyphenyl)-valeric acid | | 02 | |
| 5-(3',4',-dihydroxyphenyl)-γ-valerolactone | | 03 | |
| 5-(3',4',5'-trihydroxyphenyl)-γ-valerolactone | | 04 | |
| 5-(3',5'-dihydroxyphenyl)-γ-valerolactone | | 05 | |
| 3-Hydroxyphenylvaleric acid | | 06 | |
| **Stilbenes**  Resveratrol | | 01 | |
| Resveratrol 3-O-glucoside | | 02 | |
| 3,4,5,4'-Tetramethoxystilbene | | 03 | |
| 3'-Hydroxy-3,4,5,4'-tetramethoxystilbene | | 04 | |
| 4'-Hydroxy-3,4,5-trimethoxystilbene | | 05 | |
| 4-Hydroxy-3,5,4'-trimethoxystilbene | | 06 | |
| Resveratrol 3-sulfate | | 07 | |
| Resveratrol 3,5-disulfate | | 08 | |
| Resveratrol 3,4'-disulfate | | 09 | |
| Resveratrol 3-O-glucuronide | | 10 | |
| Resveratrol 4'-O-glucuronide | | 11 | |
| Dihydroresveratrol | | 12 | |
| **Lignans**  Secoisolariciresinol | | 01 | |
| Matairesinol | | 02 | |
| Lariciresinol | | 03 | |
| Secoisolariciresinol di-O-glucoside | | 04 | |
| 7-Hydroxymatairesinol | | 05 | |
| Enterolactone | | 06 | |
| Sesaminol 2-O-triglucoside | | 07 | |
| Sesaminol | | 08 | |
| Cyclolariciresinol | | 09 | |
| Enterodiol | | 10 | |
| 7-Hydroxyenterolactone | | 11 | |
| **Others** | |  | |
| Isopropyl 3-(3,4-dihydroxyphenyl)-2-hydroxypropanoate | 01 | |  |
| Oleuropein | 02 | |  |
| Urolithin A | 03 | |  |
| 1,3,5-Trimethoxybenzene | 04 | |  |
| 4-Ethylbenzoic acid | 05 | |  |
| 4-Ethylphenol | 06 | |  |
| Homovanillyl alcohol | 07 | |  |
| Hydroxytyrosol | 08 | |  |
| Tyrosol | 09 | |  |
| Urolithin C | 10 | |  |
| Vanilloylglycine | 11 | |  |
|  | |  | |
